# Federated, governed, and interoperable? The emerging architecture of public human genomic data infrastructures: a European perspective

**DOI:** 10.3389/fgene.2026.1819270

**Published:** 2026-04-01

**Authors:** Marco Antonio Tangaro, Matteo Chiara, Graziano Pesole, Federico Zambelli

**Affiliations:** 1 Institute of Biomembranes, Bioenergetics and Molecular Biotechnologies, National Research Council (CNR), Bari, Italy; 2 National Institute for Nuclear Physics (INFN), Section of Bari, Bari, Italy; 3 Department of Biosciences, University of Milan, Milano, Italy; 4 Department of Biosciences, Biotechnologies and Biopharmaceutics, University of Bari, Bari, Italy

**Keywords:** data governance, EHDs, federated analysis, GA4GH, GDPR, human genomic data, interoperability, trusted research environments

## Abstract

Public infrastructures for human genomic data are increasingly incorporating federated approaches alongside centralized and cloud-native models, yet operational federation remains constrained by unsolved challenges at the legal, semantic, and technical layers. We describe the current landscape along three analytical axes, taking a primarily European perspective while drawing on global examples to highlight broader trends. First, we compare architectural models, centralized archives such as the European Genome-phenome Archive (EGA) and the database of Genotypes and Phenotypes (dbGaP), cloud-native platforms for data analysis, and federated networks exemplified by the European Genomic Data Infrastructure (GDI), highlighting their specific trade-offs on scalability, sovereignty, and analytical flexibility. Second, we examine the governance layer, from the tension between the GDPR’s consent requirements and large-scale secondary use, through the European Health Data Space (EHDS) and Health Data Access Bodies, to machine-readable authorization via GA4GH Passports and the Data Use Ontology. Third, we assess interoperability and semantic alignment, including the role of GA4GH technical standards, FAIR metadata principles, and emerging schema harmonization efforts such as the German Human Genome-Phenome Archive (GHGA). We argue that the central challenge is no longer building individual platforms, but aligning heterogeneous regulatory interpretations, metadata models, and trust frameworks across jurisdictions. Addressing this alignment gap will determine whether federated genomics delivers on its promise of large-scale, privacy-preserving data reuse.

## Introduction

The cost of sequencing a human genome has dropped by several orders of magnitude since the completion of the Human Genome Project, transforming national genome programs, precision medicine, and cancer-genomics initiatives into prolific data producers (Alvarellos et al., 2023). Yet the capacity to store, integrate, and responsibly reuse these data has not kept pace. Today, petabyte-scale datasets generated across dozens of jurisdictions are governed by different legal frameworks, described by inconsistent metadata schemas, and hosted on platforms that were often designed before federation was a design requirement. The clinical translation of genomic findings further amplifies the urgency: exome and genome sequencing are now routine diagnostic tools in many healthcare systems, and their clinical value depends directly on the availability of large, well-curated reference datasets (Shickh et al., 2021; Smetana and Brož, 2022).

Historically, the field relied on centralized repositories: the NCBI database of Genotypes and Phenotypes (dbGaP) for US-funded studies and the European Genome-phenome Archive (EGA), jointly operated by EMBL-EBI and the Centre for Genomic Regulation, for European and international submissions (Mailman et al., 2007; Lappalainen et al., 2015). These archives established the controlled-access paradigm with Data Access Committees (DACs) reviewing each request against the original consent. However, they require physical data transfer to the researcher, which conflicts with emerging data-sovereignty legislation and scales poorly as datasets grow into the terabyte range. More recent developments have introduced cloud-native analysis environments and federated networks in which computation moves to the data rather than *vice versa*. Secondary use of clinical genomic data represents perhaps the most critical and contentious point in this landscape. While data generated in a diagnostic context are, in many jurisdictions, considered strictly non-reusable beyond their original clinical purpose, these same datasets are essential for improving diagnostic yield and implementing precision medicine at scale. In particular, their reuse for estimating allele frequencies and refining variant interpretation frameworks could substantially benefit the broader patient community, serving a clear collective good (Becker et al., 2024).

At the same time, the regulatory landscape has expanded considerably: for example, in Europe, the EU General Data Protection Regulation (GDPR), the Data Governance Act (DGA), and the European Health Data Space (EHDS) now coexist with domain-specific standards from the Global Alliance for Genomics and Health (GA4GH) and research-infrastructure policies from Europe’s distributed infrastructure for life-science data, ELIXIR (Saunders et al., 2019). The result is a stratified and polycentric ecosystem in which technical architecture, legal governance, and semantic interoperability, defined as the ability of different systems to interpret exchanged data with a shared, formally defined meaning, ensuring that annotations are understood equivalently across jurisdictions and infrastructures, must advance in concert if large-scale data reuse is to become routine.

We organize the landscape along three analytical axes: architectural models, governance and access control, and interoperability and semantic alignment. We argue that the central challenge has shifted from building individual platforms to aligning heterogeneous layers across jurisdictions. We draw primarily on developments of the past few years and focus on infrastructures that handle individual-level human genomic and phenotypic data under controlled or regulated access. Although genomic data-sharing initiatives are advancing globally, this analysis focuses primarily on Europe, while drawing parallels with developments in the United States, where public genomic infrastructures and regulatory frameworks are comparatively mature and federated or cloud-based architectures are being institutionalized at scale. These regions provide instructive case studies for examining how architecture, governance, and interoperability interact in practice. Where relevant, we reference additional models that offer complementary governance configurations.

## Architectural models of public genomic infrastructures

**FIGURE 1:**
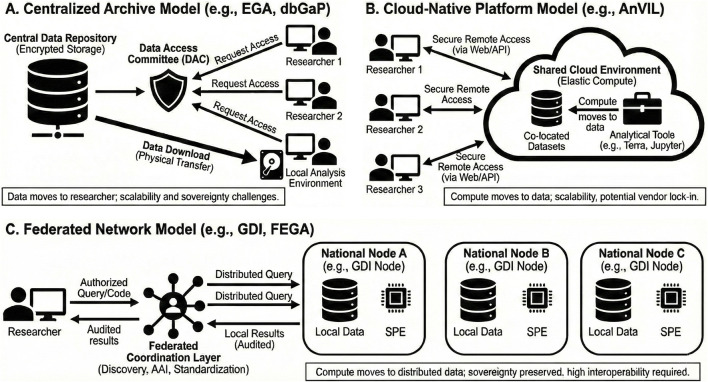
Architectural models of public genomic data infrastructures. **(A)** Centralized archive model (e.g., EGA, dbGaP), in which data are stored in a central repository and access is mediated by a data access committee; data are typically transferred to the researcher’s local environment for analysis. **(B)** Cloud-native platform model (e.g., AnVIL), where datasets and analytical tools are co-located in a shared cloud environment and researchers access them remotely, with computation moving to the data. **(C)** Federated network model (e.g., GDI, FEGA), in which data remain within national or institutional nodes and authorized queries are executed locally through Secure Processing Environments, with audited results returned through a coordination layer supporting discovery, authentication and standardization. These models are not mutually exclusive and may coexist within hybrid infrastructures. While technical interoperability has advanced across all three architectures, alignment at the legal, governance, and semantic layers remains uneven and shapes how effectively large-scale genomic data reuse can be achieved.

Public genomic data infrastructures are commonly characterized along three architectural dimensions: centralized repositories, cloud-native analysis environments, and federated coordination layers (Figure 1). Rather than representing mutually exclusive models, these components are increasingly assembled into integrated systems in which centralized metadata management, elastic cloud computing, and federated governance coexist. Different infrastructures emphasize these dimensions to varying degrees, and the effectiveness of large-scale genomic data reuse ultimately depends on how coherently these components are aligned.

The specific configuration adopted by an initiative reflects how it balances analytical flexibility, data sovereignty, and long-term sustainability. Centralized archives remain the backbone of controlled-access genomic data sharing. EGA, which surpassed 4,500 studies and millions of archived files by 2022, and at the time of writing reports more than double that figure for studies and an archive of over 20,000 Tb, stores encrypted data and distributes them to approved researchers after DAC authorization (Freeberg et al., 2022). dbGaP follows a similar model under US National Institutes of Health oversight (Tryka et al., 2014), and the Japanese Genotype-phenome Archive (JGA) serves the Asia-Pacific region (Kodama et al., 2015). The strengths of this approach are stable curation, unified metadata, established trust relationships, but are counterbalanced by two structural limitations: data must leave their jurisdiction of origin to be deposited in those repositories, creating friction with sovereignty-oriented regulation such as the GDPR; and the hosting institutions bear all storage and curation costs, a model whose long-term sustainability has been questioned as data volumes grow exponentially (Stark et al., 2025). In this respect, the United Kingdom has adopted a related but nationally embedded model through Genomics England, which operates a centralized Trusted Research Environment (TRE) where genomic and linked clinical data are accessed within a secure analysis perimeter rather than routinely exported. This configuration illustrates how sovereignty and oversight concerns can also be addressed within a single-jurisdiction framework (Samuel and Farsides, 2018).

Cloud-native platforms attempt to reduce data movement by co-locating datasets and analytical tools in shared computing environments. The NHGRI Analysis, Visualization, and Informatics Lab-space (AnVIL) hosts key US datasets, including the Centers for Common Disease Genomics (CCDG), the Centers for Mendelian Genomics (CMG), and eMERGE, on a commercial cloud provider and provides containerized workflows via Terra, Dockstore, and Jupyter-based notebooks (Schatz et al., 2022). Europe currently lacks a unified platform with such a scale of integration, but initiatives such as Laniakea, offer a Galaxy-based bioinformatics environment that can be deployed on institutional or national cloud providers, with on-demand encryption and dynamic resource provisioning, providing an open-source alternative that avoids vendor lock-in (Tangaro et al., 2020). Cloud-based infrastructures can address scalability by co-locating data and computation; however, when built on commercial hyperscaler platforms they may introduce vendor lock-in and cost-predictability challenges for publicly funded research. Cloud-native systems deployed on publicly governed or institutional cloud resources can reduce these dependencies while preserving elastic compute capabilities.

Federated networks represent the most recent and arguably most consequential architectural shift. Rather than centralizing data or hosting them in a single cloud, federation aims to keep datasets at their institutional or national origin and routes authorized queries or analysis code to each node. The European Genomic Data Infrastructure (GDI), building on the 1+Million Genomes (1+MG) declaration and its implementation project Beyond 1 Million Genomes (B1MG), is developing such a network across EU Member States, with each national node conforming to shared technical and governance standards (Schmitt et al., 2024). Operational milestones such as the GDI Starter Kit (released in 2023) illustrate the transition from conceptual federation to cross-border interoperability, providing shared software components and benchmarking environments across participating member states. Complementary initiatives, including projects aimed at developing interoperable blueprints for Trusted Research Environments at European scale (e.g., EOSC-ENTRUST), further signal the institutionalization of federated access models beyond pilot implementations.

The federated European Genome-phenome Archive (FEGA) extends this model by allowing national EGA instances to manage local data submissions and access while remaining discoverable through the central EGA search functions (Freeberg et al., 2022). Underlying many of these federated services is ELIXIR, Europe’s distributed research infrastructure for life-science data, which provides or contributes to critical components such as the Authentication and Authorization Infrastructure (AAI), shared compute resources, and interoperability standards on which national nodes depend (Harrow et al., 2021). Privacy-enhancing technologies such as differential privacy, secure multi-party computation, homomorphic encryption, and federated learning can, in principle, complement this architecture by offering cryptographic or statistical guarantees against re-identification, although their computational overhead and integration complexity limit routine deployment (Rujano et al., 2024).

In practice, these categories are not mutually exclusive. A researcher may discover a dataset through the EGA Beacon (centralized metadata), obtain authorization via a national FEGA node (federated governance), and execute the analysis on a cloud-hosted Secure Processing Environment (SPE) – a controlled technical and organizational environment designed to ensure compliance with applicable EU law. The emerging pattern is therefore best described as a hybrid architecture, in which centralized discovery, federated governance, and elastic computation are layered. The key unresolved question is whether these layers can be standardized sufficiently to function as a coherent system.

An alternative response to this alignment challenge is to embed harmonization at the point of data generation. The recently launched Genome of Europe (GoE) initiative exemplifies this approach: it seeks to build a pan-European reference genomic database by sequencing and integrating genomes from representative European populations under shared ethical, legal, and technical conditions, reducing fragmentation of genomic information and aligning with broader efforts such as the 1+Million Genomes Initiative and the Genomic Data Infrastructure project (Caporale et al., 2025). By integrating data production within a coordinated framework of standards and governance objectives, such projects could lessen downstream semantic divergence and enhance the utility of large cross-border genomic analyses.

## Governance and access: from consent to machine-readable authorization

If architecture determines where data reside and how they are computed, governance determines who may access them, for what purpose, and under what conditions. The governance of genomic data is shaped by the interaction of privacy law, research ethics, and emerging sector-specific regulation. While these frameworks play a critical role in safeguarding individuals’ rights and public trust, their heterogeneous implementation across jurisdictions introduces operational complexity and coordination challenges.

The GDPR classifies genomic sequences as “special category” data under Article 9, permitting processing only under enumerated legal bases such as explicit consent, substantial public interest, or scientific research with appropriate safeguards (Shabani and Borry, 2018). In practice, the tension between the GDPR’s specificity-of-purpose requirement and the open-ended nature of research has generated divergent national interpretations. Recital 33 allows broader consent for scientific research, but its interpretation and implementation vary across Member States: some require ethics-committee approval for each secondary use, whereas others accept a single broad-consent instrument covering a wide range of future research (Christofidou et al., 2025). In addition, Article 89 of the GDPR allows Member States to introduce specific safeguards and derogations for scientific research, contributing to the heterogeneous national interpretations that shape current genomic data-sharing practices. This heterogeneity has direct operational consequences; the same dataset may be shareable within one jurisdiction and legally immovable in another, fragmenting multi-site studies at the governance layer even when the technical infrastructure is in place (Royo et al., 2025). Outside Europe, the regulatory context differs. In the United States, privacy protections for genomic data are distributed across instruments such as the HIPAA Privacy Rule and the Common Rule, which apply to specific categories of entities and research activities. This sectoral structure creates a regulatory landscape that differs markedly from the comprehensive approach of the GDPR (Casaletto et al., 2023).

Two recent EU instruments are designed to reduce fragmentation. The Data Governance Act (DGA, in force since 2023) introduces “data altruism,” a voluntary mechanism through which individuals can make personal data available for research of general interest, potentially complementing traditional consent with a broader social mandate (Christofidou et al., 2025).

More consequentially, the European Health Data Space (EHDS), adopted in 2025, mandates that each Member State establish a Health Data Access Body (HDAB) responsible for issuing data permits for secondary use (Svingel et al., 2025). Within this framework, it is important to distinguish between *data holders*–entities such as hospitals, biobanks, or sponsors responsible for making datasets available in interoperable formats–and *data users*, namely, researchers or institutions requesting access through data permits.

Although the EHDS Regulation entered into force in 2025, its provisions for secondary use are subject to a phased implementation. In particular, categories such as genomic and clinical-trial data are expected to fall under full applicability only after an extended transitional period. During this interval, cross-border genomic data sharing will likely continue to rely primarily on national GDPR-based frameworks, creating a phase of normative latency between technical federation and legal harmonization. Furthermore, the introduction of opt-out mechanisms and the possibility for member states to maintain additional safeguards for genetic data introduce potential structural asymmetries. Divergent opt-out rates or consent requirements may translate into uneven data availability across national nodes, with potential implications for statistical power, representativeness, and bias in cross-border genomic analyses. Furthermore, the EHDS poses targeted implementation challenges: consent-model heterogeneity across member states, the computational demands of large sequencing datasets within SPEs, and the as-yet unresolved interface between the EHDS framework and different national privacy legislations, which may introduce additional and heterogeneous constraints beyond the GDPR (Royo et al., 2025).

At the technical-governance interface, GA4GH has developed machine-readable instruments that encode access conditions into interoperable standards. The Data Use Ontology (DUO) translates consent clauses into structured, queryable terms, enabling automated matching between a dataset’s permitted uses and a researcher’s stated purpose (Rehm et al., 2021). GA4GH Passports extend this logic to researcher identity: a Passport bundles digitally signed assertions such as institutional affiliation, ethics approval, and prior authorization into a portable credential that can be verified by any compliant data repository, eliminating redundant application processes across multiple archives (Voisin et al., 2021). Together, DUO and Passports form the backbone of a governance-aware data access layer that aims to make authorization decisions computable and portable across federated nodes. Thus, beyond defining technical standards, GA4GH has increasingly focused on implementation guidance and certification criteria, reflecting a shift from conceptual interoperability to validated, production-level deployments within research and healthcare infrastructures.

However, the governance space remains contested (Graham et al., 2023). Have argued that operational models designed to support secure data sharing and analysis in clinical and research settings such as, for example, Trusted Research Environments (TREs), might function primarily by reducing the need for trust: they contain data within controlled perimeters and shift oversight from relational accountability to technical containment. This critique surfaces a broader tension in the field, whether governance should be grounded in interpersonal and institutional trust relationships or in auditable, technology-enforced controls (Thaldar, 2025). Offers a middle-ground conceptual framework with the Seven-Dimensional Data Visiting Framework (7D-DVF), which disaggregates federated access into adjustable dimensions such as researcher autonomy, data visibility, output governance, and auditability, allowing proportional, context-sensitive configurations rather than binary open-or-closed decisions. This approach would reframe federated access not as a monolithic model but as a set of adjustable governance parameters.

A further unresolved dimension concerns accountability and liability in federated systems. When data access decisions are distributed across national nodes, and authorization workflows are mediated by machine-readable instruments such as DUO and GA4GH Passports, responsibility becomes stratified and potentially ambiguous. In the event of a data breach, misinterpretation of use restrictions, or unlawful secondary processing, it may be unclear whether liability rests with the data holder, the federated coordination layer, the authentication provider, or the researcher’s host institution (Beyvers et al., 2026). Legal analyses highlight that federated learning consortia must navigate complex governance and data-protection requirements, including joint controller agreements and GDPR compliance (Malpetti et al., 2025; Lieftink et al., 2024). Federated architectures therefore require not only interoperable technical standards but also clearly articulated responsibility frameworks, including audit trails, traceable authorization chains, and harmonized enforcement mechanisms across jurisdictions. Without such clarity, federation risks diffusing responsibility precisely when cross-border governance demands greater institutional accountability.

## Interoperability and semantic alignment

Architecture and governance define the physical and legal topology of human data sharing; interoperability determines whether datasets from different nodes can actually be combined for scientific analysis. Three layers of interoperability are relevant: protocol-level (how systems communicate), semantic (what the metadata mean), and policy-level (whether use conditions are interpretable across borders).

At the protocol level, GA4GH has established a mature technical stack. For example, the Beacon API enables variant and phenotypic discovery across federated nodes. Its original version (v1) implemented binary presence/absence queries to minimize data exposure; however, membership-inference attacks demonstrated that even such constrained interfaces cannot eliminate re-identification risk entirely (Von Thenen et al., 2019). Beacon v2 substantially extends the protocol with richer query types, including case-level and genomic-variant-level requests, and supports tiered access controls and handover mechanisms to established clinical data standards such as Phenopackets and FHIR (Rambla et al., 2022). These extensions enable more expressive discovery while allowing implementers to calibrate response granularity to the sensitivity of the underlying data. Yet evidence suggests that even advanced protocol safeguards cannot completely neutralize re-identification risks in high-dimensional genomic data (Saleem et al., 2025). This limitation underscores the need for complementary legal and organizational protections alongside technical design.

The *htsget* protocol provides standardized streaming access to alignment and variant-call files, while the Tool Registry Service (TRS) and Workflow Execution Service (WES) allow reproducible analysis pipelines to be shared and executed across heterogeneous compute environments (Rehm et al., 2021). These standards have been adopted by GDI, FEGA, and several national genome initiatives, creating a *de facto* communication layer for a European genomic data infrastructure.

Semantic interoperability remains a harder and less mature requirement. Human genomic datasets carry rich phenotypic, clinical, and sample-level metadata whose encoding varies widely across institutions and countries: e.g., different ontologies for disease classification, non-standardized sample identifiers, and inconsistent conventions for consent-related annotations. The German Human Genome-Phenome Archive (GHGA) has recently documented a systematic effort to align its metadata schema with the EGA model and other domain-relevant standards, including GA4GH specifications such as DUO and Phenopackets, identifying both convergences and persistent gaps in terminology and granularity (Mauer et al., 2026). These interoperability efforts unfold within a broader normative framework shaped by the FAIR principles. However, in the context of sensitive human data, FAIR cannot be equated with unrestricted openness. Different types of genetic and omics data carry varying levels of re-identification risk: sequencing-based assays with high genome coverage generally increase the risk of re-identification, whereas targeted genotyping or lower-resolution expression assays (e.g., microarrays or targeted panels) provide sparser data and may pose lower re-identification risk (Thomas et al., 2024). Operationalizing FAIR in this domain therefore requires a controlled-access model in which metadata remain as open and standardized as possible, while individual-level data are subject to governance mechanisms proportionate to their identifiability risks (Joly et al., 2016).

Policy interoperability, that is, the machine readability of access conditions across jurisdictions, bridges governance and semantics. DUO codes attached to a dataset in Germany must be interpretable by a Passport-verification service in Finland; an HDAB data permit issued under the EHDS must map onto the access-control logic of a national SPE. Achieving this requires not only shared ontologies but also mutual recognition of governance decisions, a challenge that is institutional and political as much as technical. The federated infrastructure model amplifies the problem: whereas a centralized archive can impose a single metadata standard, a federation of autonomous nodes must negotiate consensus without coercion (Harrow et al., 2021).

A federation does not eliminate the burden of data harmonization; rather, it redistributes it. In centralized repositories, metadata normalization occurs primarily at the point of ingestion. In federated systems, by contrast, each node retains responsibility for local data curation while simultaneously aligning with shared ontologies, schemas, and access vocabularies. This distributed model increases coordination demands and requires sustained investment in semantic governance, tooling, and human expertise. The long-term viability of federated genomics will therefore depend not only on technical interoperability standards, but also on durable mechanisms for metadata stewardship, version control, and cross-node schema evolution (Rehm et al., 2021). Without sustained semantic coordination, federation risks producing technically connected yet analytically incompatible datasets (Saunders et al., 2019). Until protocol, semantic, and policy interoperability converge, the promise of seamless cross-border federated analysis will remain aspirational.

## Discussion: unresolved tensions and future directions

The preceding analysis reveals a field in transition. Public human genomic data infrastructures are moving beyond proof-of-concept federations and are now on the path to implementing continent-scale networks such as GDI. Yet several unresolved tensions constrain the ecosystem and will shape its trajectory in the coming years.

The first is a sustainability gap. Centralized archives, cloud platforms, and federated nodes alike require sustained investment in infrastructure, governance, and technical expertise. Stark et al. (2025) emphasize that scaling human genomic data sharing demands coordinated policy support and long-term infrastructure funding that extend beyond the duration of individual projects. The EHDS offers a partial answer by embedding data access in a regulatory framework that implies ongoing national commitment, but the operational budgets of HDABs and SPEs remain undefined. Without institutional permanence, the metadata curation and standard maintenance that federations depend on could be eroded over time. A further structural development in this direction is the introduction of the European Digital Infrastructure Consortium (EDIC), a legal instrument designed to support the establishment of intergovernmental digital infrastructures with long-term mandates. By allowing member states to jointly create and fund cross-border infrastructures under a dedicated EU framework, the EDIC model provides a potential alternative to time-limited project funding and may strengthen institutional continuity. In the context of genomic data infrastructures, EDIC status could serve as a governance anchor for initiatives such as GDI or related federated services, aligning technical interoperability with sustained political commitment and shared financial responsibility. Whether EDIC will be effectively leveraged to consolidate federated genomics into a durable European public infrastructure remains an open but strategically significant question.

A second tension lies between regulatory harmonization and national sovereignty. While the EHDS establishes a framework for cross-border secondary use, Member States retain discretion in the implementation of key safeguards, particularly for genetic and genomic data, and in the interpretation of consent and ethical requirements. As highlighted by Royo et al. (2025), this may result in a system that is technically interoperable yet legally fragmented, where similar data access requests can receive different outcomes depending on the jurisdiction involved. Moving from protocol interoperability to governance interoperability, such as mutual recognition of data permits and alignment between machine-readable use restrictions and regulatory categories, represents the next frontier and will require sustained regulatory alignment and intergovernmental coordination, in addition to technical standardization.

Another emerging tension concerns the role of commercial actors within publicly governed genomic infrastructures. Pharmaceutical companies, biotechnology firms, and digital health providers increasingly rely on large-scale genomic datasets for translational research and innovation. While the EHDS permits secondary use for innovation purposes, member states retain discretion in defining safeguards, access conditions, and fee structures for commercial users. As federated systems mature, they will need to articulate transparent and consistent access policies that balance public trust, equitable benefit sharing, and the promotion of innovation. Clarifying the governance frameworks under which commercial entities may access federated genomic resources will be critical to sustaining institutional legitimacy and ensuring that publicly contributed data support broadly distributed societal benefits.

The last tension concerns trust itself. The field is investing heavily in technical containment via TREs and SPEs, but, as Graham et al. (2023) argue, such models reduce the need for trust by substituting technical control for relational vulnerability. A governance-aware ecosystem will therefore require mechanisms that extend beyond perimeter security: robust auditability, transparent access processes, and governance arrangements capable of sustaining public trust and equitable benefit sharing (Thaldar, 2025; Alvarellos et al., 2023). These governance challenges intersect with potential inequities in global genomics. Clinical-genomic datasets and the infrastructures that host them remain disproportionately concentrated in high-income countries, limiting representation and potentially constraining the distribution of downstream benefits. Ensuring that federated architectures enhance, rather than merely replicate, existing participation patterns will require deliberate design choices that prioritize inclusion alongside technical interoperability.

In conclusion, public genomic data infrastructures increasingly integrate federated and governance-aware architectures, alongside centralized and cloud-based components, yet progress at the technical layer has advanced more rapidly than alignment at the legal and semantic levels. The field’s next challenge is therefore not simply to build additional platforms, but to close the alignment gap between architecture, regulation, and metadata, transforming diverse national and regional nodes into a coherent, trustworthy, and sustainable system for large-scale genomic data reuse.
